# PSMA PET/CT imaging and its application to prostate cancer treatment

**DOI:** 10.1007/s11604-024-01646-9

**Published:** 2024-09-03

**Authors:** Tomoaki Otani, Ryusuke Nakamoto, Shigeaki Umeoka, Yuji Nakamoto

**Affiliations:** 1 Department of Diagnostic Radiology, Japanese Red Cross Society Wakayama Medical Center, 4-20 Komatsubara-dori, Wakayama, 640-8558 Japan; 2 Department of Diagnostic Imaging and Nuclear Medicine, Graduate School of Medicine, Kyoto University, Kyoto, Japan; 3 Preemptive Medicine and Lifestyle Related Disease Research Center, Kyoto University Hospital, Kyoto, Japan

**Keywords:** Prostate-specific membrane antigen (PSMA), Oligometastasis, Salvage radiation therapy (SRT), Metastasis-directed therapy (MDT)

## Abstract

Recognition of the importance of prostate-specific membrane antigen (PSMA) PET/CT in the diagnosis of prostate cancer has steadily increased following the publication of extensive data on its diagnostic accuracy and impact on patient management over the past decade. Several recent clinical trials and investigations regarding PSMA PET/CT have been ongoing in our country, and this examination is expected to become increasingly widespread in the future. This review explains the characteristics of PSMA PET/CT, its diagnostic capabilities and superiority over other modalities, the three proposed PSMA PET/CT interpretation criteria (the European Association of Nuclear Medicine [EANM], the Prostate Cancer Molecular Imaging Standardized Evaluation [PROMISE], and the PSMA Reporting and Data System [PSMA-RADS]), and the application of PSMA PET/CT to prostate cancer treatment (improvement of local control, irradiation of oligometastases, and salvage radiotherapy), incorporating actual clinical images and the latest findings.

## Introduction

Prostate cancer is one of the most common cancers worldwide, and its incidence has been increasing in recent years [1–3]. Cancer statistics in Japan have shown prostate cancer as the leading cause of cancer among men [4]. Prostate cancer is classified into three categories based on the stage of progression: localized prostate cancer, locally invasive prostate cancer, and metastatic prostate cancer [5]. In the management of localized prostate cancer, the D'Amico classification and National Comprehensive Cancer Network (NCCN) risk classification are occasionally used [5, 6], categorizing the disease from low to high risks based on Gleason score, T stage, and concentration of prostate-specific antigen (PSA). Around 74% of prostate cancers are localized at the time of diagnosis, allowing effective treatment with surgery and radiation therapy [3]. The prognosis for these localized cases is relatively good, with a 5-year relative survival rate of approximately 99% in Japan [4]. However, biochemical recurrence (BCR) is not uncommon after curative treatment, particularly in high-risk groups, occurring in 40% after total resection and 36% after intensity-modulated radiation therapy (IMRT) [7, 8]. Conventional imaging modalities, such as CT, MRI, and bone scintigraphy, are insufficient to detect early recurrence or small metastases. Prostate-specific membrane antigen (PSMA) is a protein that is abundantly expressed in prostate cancer, particularly in castration-resistant prostate cancer [9]. PSMA PET/CT has emerged as a useful modality for planning the treatment of prostate cancer and is expected to significantly impact the management of prostate cancer due to its high lesion-detection ability, particularly in cases of recurrent prostate cancer. Recently, several clinical trials and investigations regarding PSMA-PET/CT have been ongoing in our country, but this imaging modality has not yet been approved. In this paper, we provide an overview of the utility of PSMA-PET/CT scans and key considerations for interpretation, presenting actual case studies and referring to the literature. We also discuss the application of PSMA-PET/CT to prostate cancer treatment.

## Basic knowledge of PSMA

PSMA, also known as glutamate carboxypeptidase II, is a type II transmembrane protein. This enzyme is frequently overexpressed in prostate cancer, whereas expression in normal prostate is low. Expression of PSMA is progressively increased in castration-resistant prostate cancer and metastatic lesions [9, 10]. PSMA may thus represent a promising target for prostate cancer treatment. Labeling PSMA-ligand with radioactive isotopes facilitates both imaging and internal irradiation. The localization of the catalytic site in the extracellular domain of PSMA has enabled the development of small, highly specific inhibitors [9, 10]. Ligand binding to PSMA is internalized via endocytosis into cells, leading to the deposition and retention of ligands in the tumor. This process results in high image quality for diagnosis and a high local dose for internal irradiation, with rapid clearance from the blood and normal soft tissues [9]. The European Association of Urology guidelines states that PSMA PET/CT is more accurate than CT and bone scan for staging high-risk disease (evidence level 1b), and that PSMA PET should be offered to men showing a persistent PSA > 0.2 ng/ml, if the result is expected to influence subsequent treatment decisions (weak recommendation) [11, 12]. A variety of ^68^ Ga- and ^18^F-labeled PSMA-targeted diagnostic agents have been developed to date. Table 1 illustrates the characteristics of ^68^ Ga and ^18^F, along with examples of representative agents. As For the ^68^ Ga-labeled agent, it is feasible to transition to treatment by labeling with ^177^Lu and ^225^Ac, utilizing the same chemical structure. This approach, wherein the process moves from nuclear medicine imaging results to treatment with therapeutic agents that accumulate in lesions via the same mechanism, is referred to by the coined term "theranostics," which combines therapeutics and diagnostics. The United States Food and Drug Administration (FDA) approved ^68^ Ga-PSMA-11 and ^18^F-DCFPyL in 2020 and 2021, respectively [13]. The most commonly used agent is ^68^ Ga-PSMA-11, which was introduced by the German Cancer Research Centre [14]. Particularly, in the United States, ^18^F-DCFPyL is also widely used. Another diagnostic agent, ^18^F-PSMA-1007, has recently been approved in some European countries and is known for being primarily excreted through the hepatobiliary system [15]. This offers the advantage of low urinary excretion and superior detection of lesions along the urinary tract. Another agent, ^18^F-FSU-880, was developed by Kyoto University and exhibits high binding affinity for PSMA and a favorable pharmacokinetic profile, with significant accumulation in PSMA-expressing tumors [16, 17].

**Table 1:** The characteristics of ^68^ Ga, ^18^F and representative agents

	Characteristic	Representative agents	Characteristic
^68^ Ga	- Generated by generator or cyclotron - Physical half-life: 68 min - Possible to expand into treatment by labeling with ^177^Lu and ^225^Ac, using the same chemical structure (so-called “Theranostics”)	^68^ Ga-PSMA-11	- Most widely used - The first FDA*^1^-approved agent - Indicated for diagnosing and staging of PC^*2^, and for detecting site of metastasis for biochemical recurrence after curative treatment
		^68^ Ga-PSMA-I&T	- Comparable results to ^68^ Ga-PSMA-11 regarding the detection rate of PC*^2^ - Slightly higher uptake in blood pool, bone marrow and background due to slower clearance compared to ^68^ Ga-PSMA-11
^18^F	- Generated by cyclotron - Physical half-life: 110 min - Higher spatial resolution due to a shorter positron range - Improved commercial availability due to a longer half-life	^18^F-DCFPyL	- Another FDA*^1^-approved agent - Comparable tumor-to-background contrast compared to ^68^ Ga-PSMA-11
		^18^F-PSMA-1007	- Minimum excretion in the urine resulted in improving small lesions adjacent to the urinary tract - Nonspecific bone uptake was reported (Ribs most frequent)
		^18^F-FSU-880	- Has seven times affinity for PSMA compared to ^18^F-DCFPyL

*1 food and drug administration, *2 prostate cancer

## Ability of PSMA PET/CT to detect recurrent prostate cancer

Recurrences after the radical treatment of prostate cancer are primarily determined based on changes in PSA levels. Following total prostatectomy, BCR is defined as an increase in PSA level by ≥ 0.2 ng/ml between two consecutive measurements taken 2–4 weeks apart [18]. After IMRT, BCR is defined by an increase of ≥ 2.0 ng/ml from the lowest post-treatment PSA value [19]. In some studies, the conventional imaging modalities such as CT and bone scintigraphy identified recurrent lesions in only 30% of intermediate-risk patients and 46% of high-risk and very high-risk patients following IMRT [7, 20]. In more than half of all those cases, recurrent lesions remain undetected, suggesting that the conventional modalities may be insufficient.

**Fig. 1:**
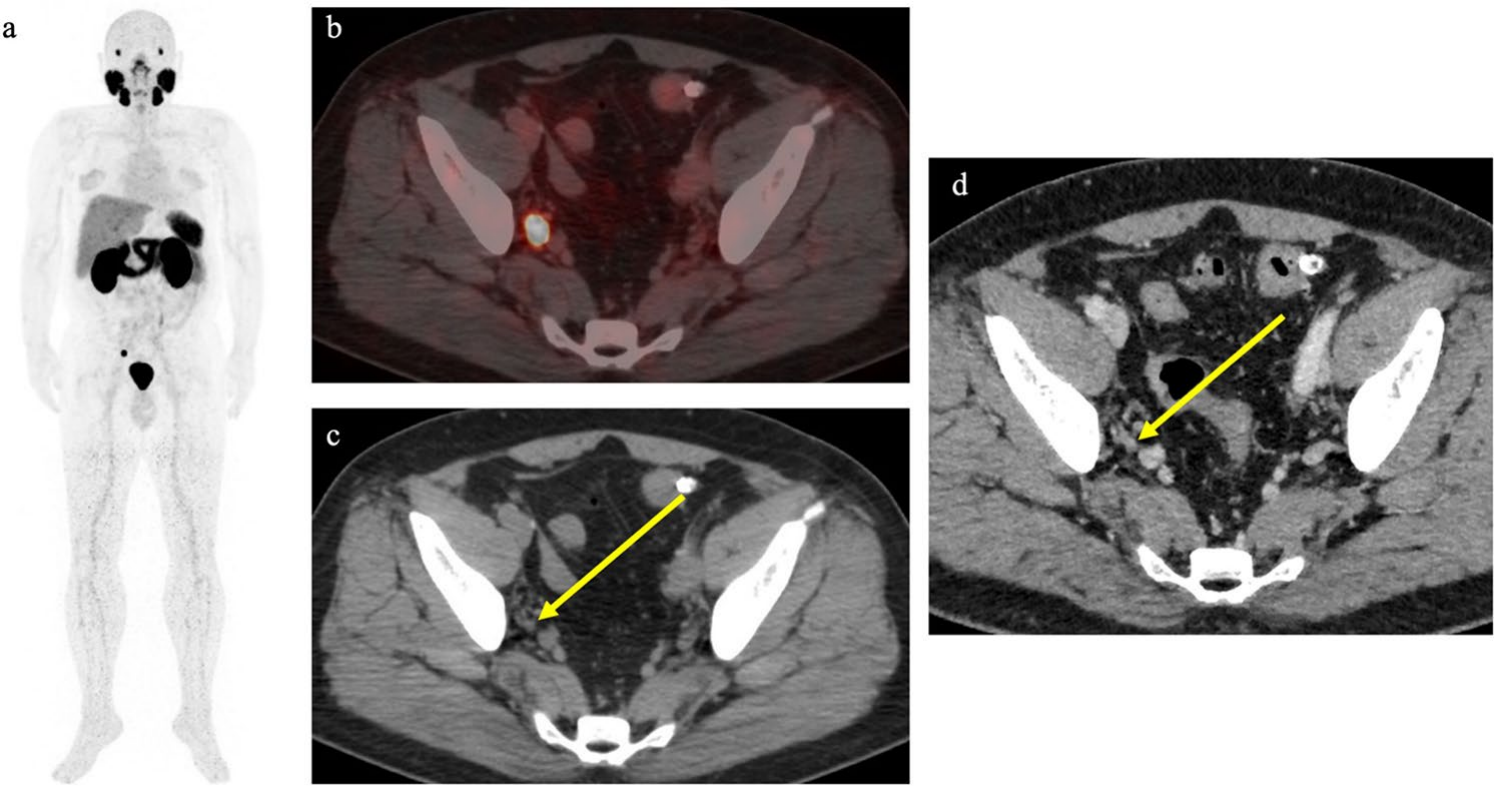
PET/CT using ^68^ Ga-PSMA-11 in a 65-year-old man with biochemical recurrence following total prostatectomy (pT3aN1, Gleason score 4 + 5). PSA level increased to 1.35 ng/ml. Focal uptake was observed in a 6-mm lymph node in the right obturator region (arrows), suggesting lymph-node metastasis. **a** Maximum intensity projection image. **b** Transaxial PET/CT fusion image. **c** Transaxial CT. **d** Transaxial contrast-enhanced CT

**Fig. 2:**
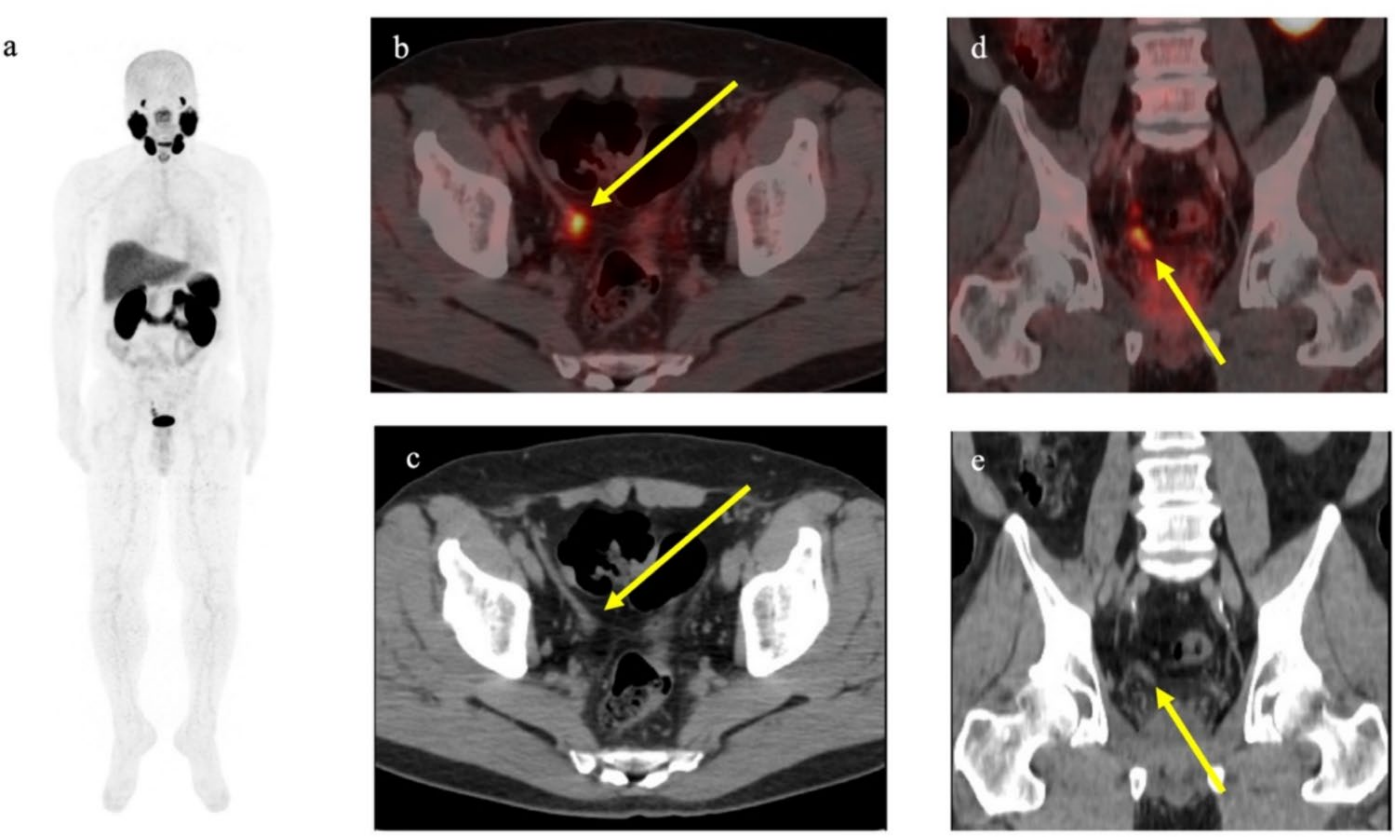
PET/CT using ^68^ Ga-PSMA-11 in a 71-year-old man with biochemical recurrence after radiation therapy (cT3aN0, Gleason score 4 + 5). PSA level increased to 8.20 ng/ml. Focal uptake was observed from the right seminal vesicle to the vas deferens, with no other significant uptake apparent, suggesting metastasis. Combined androgen blockade therapy was administered and the PSA level decreased to 0.04 ng/ml. **a** Maximum intensity projection image. **b** Transaxial PET/CT fusion image. **c** Transaxial CT. **d** Coronal PET/CT fusion image. **e** Coronal CT

A significant advance in detection methods came with ^68^ Ga-PSMA-11. In a large study including around 2500 patients with recurrence after total prostatectomy, detection rates were 43%, 58%, 72%, 84%, and 91% for PSA levels of ≤ 0.2 ng/ml, 0.2–0.5 ng/ml, 0.5–1.0 ng/ml, 1.0–2.0 ng/ml, and ≥ 2.0 ng/ml, respectively [21]. PSMA-targeted tracers labeled with ^18^F show a shorter positron range and improved spatial resolution compared to ^68^ Ga-labeled PSMA-targeted tracers. As a result, they have a higher lesion-detection capacity, particularly in patients with low PSA values [22]. Studies using ^18^F-DCFPyL PET/CT and ^18^F-PSMA-1007 have reported detection rates of 48–89% in patients with PSA levels ≤ 0.5 ng/ml [22–25]. Notably, lesions were identified in more than half of patients with PSA levels as low as 0.5 ng/ml, which had been challenging to detect in the past. One reason for the superiority of PSMA PET/CT in comparison to the conventional imaging modalities is the ability to detect small lesions. The efficacy of PSMA PET/CT in detecting small lesions was highlighted in a study where lymph-node dissection was performed in 30 patients with suspected lymph-node metastases detected by PSMA PET/CT following BCR. A nearly 100% positive predictive value was shown, with approximately 90% negative predictive value and about 95% accuracy [26]. The average size of metastases in positive cases was 7.5 mm, below the general size criterion for detectability. However, the negative predictive value was about 90%, and lymph-node metastases with an average size of 4 mm were generally missed, representing false-negative results, indicating challenges in detecting lesions below a certain size. Figure 1 shows a case of small lymph-node metastasis revealed by PSMA PET/CT. In addition, PSMA PET/CT has also been reported to identify metastasis in unexpected sites, such as the brain, adrenal glands, penis, and orbit [27]. Figure 2 shows a case of recurrence observed in the right seminal vesicle and extending to the vas deferens. This lesion was difficult to identify with other modalities, similar to another case [28].

As for the initial tumor, the higher the Gleason score, the higher the detection rate [29]. In addition, some prospective investigations found that PSMA PET/CT changed the course of treatment in 64–68% of patients after BCR, indicating a significant impact on the selection of treatment options [30, 31].

## Comparison of PSMA PET/CT with other modalities

**Fig. 3:**
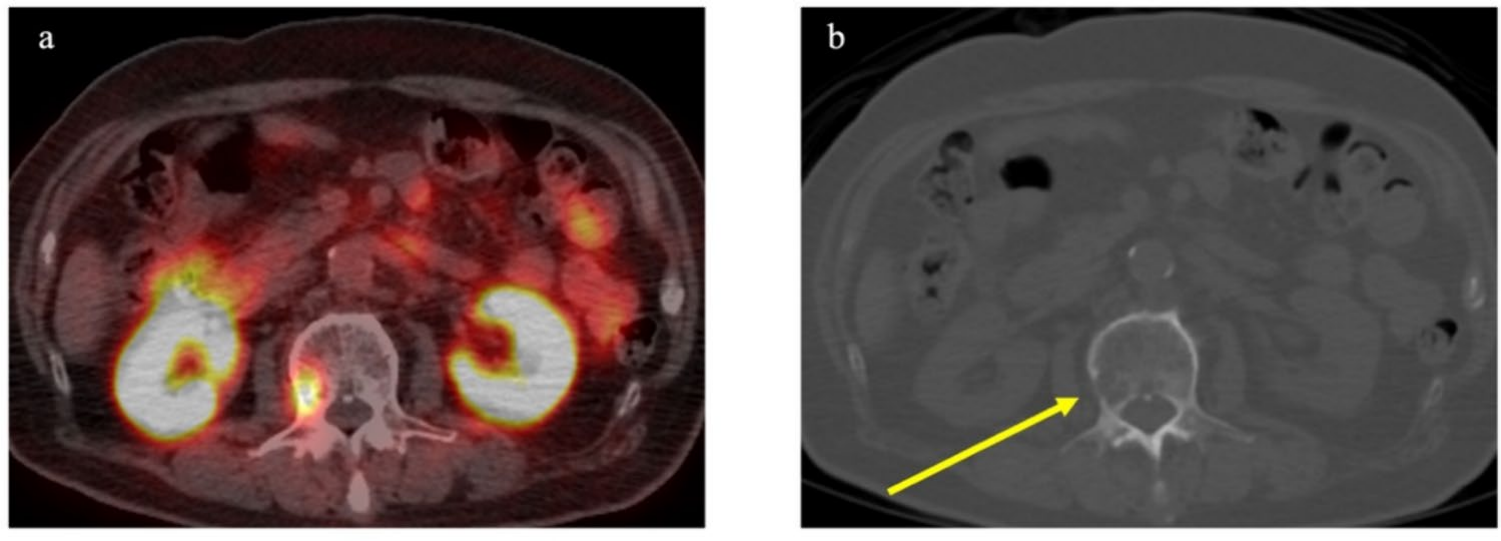
An 80-year-old man who had previously been treated with radiation therapy (cT3bN1, Gleason score 4 + 5) demonstrated biochemical recurrence. PSA level elevated to 4.46 ng/ml. PET/CT using ^18^F-FSU-880 showed focal uptake in a primarily osteolytic lesion of the lumbar spine (SUVmax: 9.4). **a** Transaxial PET/CT fusion image. **b** Transaxial CT

PSMA PET/CT has demonstrated superior capability for detecting bone metastases compared to bone scintigraphy [32–34]. A meta-analysis investigated comparisons between ^99m^Tc-bone scintigraphy and ^68^ Ga-PSMA-11 for detecting bone metastases, providing pooled sensitivity and specificity of 98% and 97%, respectively, for ^68^ Ga-PSMA-11, compared to 83% and 68%, respectively, for ^99m^Tc-bone scintigraphy. Moreover, ^68^ Ga-PSMA-11 detected bone metastases in 22.3% of cases showing negative results from ^99m^Tc-bone scintigraphy [33]. Small lesions and non-sclerotic metastases that are difficult to detect on bone scintigraphy can be detected with PSMA PET/CT. Figure 3 illustrates a case of osteolytic bone metastasis in the lumbar spine, detected by PSMA PET/CT in a patient with prostate cancer in a BCR setting.

In the assessment of the primary tumor, multiparametric MRI remains the standard modality for evaluating clinically significant prostate cancer (csPCa). Standardization using the Prostate Imaging–Reporting and Data System (PI-RADS) is widespread [35]. The combination of PSMA PET/CT with multiparametric MRI recently improved the diagnostic performance for csPCa. Emmett et al. reported that combined PSMA PET/CT and multiparametric MRI offered an improved negative predictive value (91%) compared with multiparametric MRI alone (72%) [36]. Emmett et al. also proposed a 5-point scoring system called the PRIMARY score, which utilizes pattern information and degree of uptake. That system was shown to be superior to PI-RADS in diagnostic performance, achieving an area under the curve (AUC) of 0.89 compared to 0.76 with PI-RADS [37]. Table 2 summarizes the PRIMARY score. However, false-positive uptake of PSMA PET/CT in the transitional zone due to overexpression of PSMA in non-tumoral tissues of the prostate has been reported and warrants careful assessment [38]. PSMA PET/MRI has demonstrated superior diagnostic accuracy for the localization of prostate cancer when compared to both multiparametric MRI and PSMA PET imaging alone [39–41]. This hybrid imaging modality combines the enhanced specificity of PET with the superior anatomical resolution and tumor localization provided by MRI, offering a more comprehensive approach to prostate cancer diagnosis.

**Table 2:** Summary of PRIMARY scores, a 5-point scoring system for interpreting primary lesions

Score	Pattern and intensity
1	No dominant intraprostatic pattern on PSMA. Low-grade activity
2	Diffuse transition zone activity or symmetrical central zone activity that does not extend to the prostate margin on CT (included diffuse transition zone activity that is not well above background transition zone activity)
3	Focal transition zone activity visually twice above background transition zone activity
4	Focal peripheral zone activity (any focal activity in the peripheral zone is considered abnormal)
5	Very high intensity (PSMA SUVmax > 12)

FDA-approved ^11^C-cholline PET/CT has also been widely used in PC patients in the BCR setting, but PSMA PET/CT has been found to offer superior diagnostic performance [42, 43]. According to studies that investigated diagnostic performance in BCR patients, only ^68^ Ga PSMA PET/CT detected recurrent lesions in 25% of patients, compared to only 3% detection with ^11^C-choline PET/CT, while additionally offering a better tumor-to-background ratio [43].

## Image interpretation criteria for PSMA PET/CT

PSMA PET/CT is emerging as a pivotal imaging modality in prostate cancer treatment, necessitating a standardized methodology for image interpretation as adoption of this method broadens. Three different standards have been proposed for reading PSMA PET/CT: the European Association of Nuclear Medicine (EANM) criteria [44]; the Prostate Cancer Molecular Imaging Standardized Evaluation (PROMISE) criteria [45, 46]; and the PSMA Reporting and Data System (PSMA-RADS) [47]. These criteria are detailed in Table 3. The EANM criteria defined increased uptake exceeding background levels and not typically associated with physiological uptake as anomalous. Further, all anomalous findings suggestive of recurrent prostate cancer based on clinical and imaging characteristics were classified as pathologic, and the increased uptake attributable to other diseases was described as not pathologic. The PROMISE criteria categorized uptake into four levels using comparisons with the uptake seen in the blood pool, liver, and salivary gland. The PSMA-RADS framework classified uptake into benign, likely benign, equivocal, highly likely, and almost certainly present. Crucially, the final diagnosis should be determined in combination with findings from the other modalities like CT or MRI, the location of uptake, and clinical context. In the PROMISE criteria, according to the prior probability obtained from MRI or CT findings or location of uptake, the evaluation for uptake has to be changed. That is to say, in sites typical for prostate cancer metastasis such as pelvic lymph nodes, even a PSMA score of 1 (between the levels of the blood pool and liver) should be interpreted as positive [45, 46], while uptake in atypical sites for metastatic prostate cancer should be evaluated with caution. The PSMA-RADS recommended that lesions with equivocal uptake that could not be biopsied or practically evaluated using other modalities should be assessed with follow-up after 3–6 months using PSMA PET/CT to evaluate progression of uptake [47]. Toriihara et al. examined inter- and intra-reader agreement for these three criteria, finding generally good inter- and intra-reader reproducibility [48]. However, they also noted that inter-reader disagreement most frequently arose in evaluating distant metastases using PSMA-RADS, especially for evaluating lung nodules.

**Table 3:** Overview of the three criteria for interpretating PSMA PET/CT images

	EANM*1	PROMISE*2	PSMA-RADS*3
The assessment of uptake	Focal uptake higher than adjacent background and not physiologic is regarded as “anomalous”	Four-point scale 0: below blood pool 1: Equal to or above blood pool and lower than liver 2: Equal to or above liver and lower than parotid grand 3: Equal to or above parotid grand	Five-point scale 1. Benign 2. Likely benign 3. Equivocal 4. Highly likely 5. PC*4 almost certainly present
Suspicious for malignancy	As above and below	Score 2 or higher	Score 4 or higher
Special notes	All findings suggestive of recurrent prostate cancer, based on a combination of clinical and imaging characteristics, are noted as “pathologic”	Propose miTNM framework to standardized reporting system for PSMA-ligand PET/CT	PSMA-RADS-3 indicates that additional work-up or follow-up imaging might be beneficial to better characterize the finding

*1 The European Association of Nuclear Medicine, *2 The prostate cancer molecular imaging standardized evaluation, *3 The PSMA reporting and data system, *4 prostate cancer

## Application to prostate cancer treatment

### Detection of locally recurrent disease after radiation therapy

**Fig. 4:**
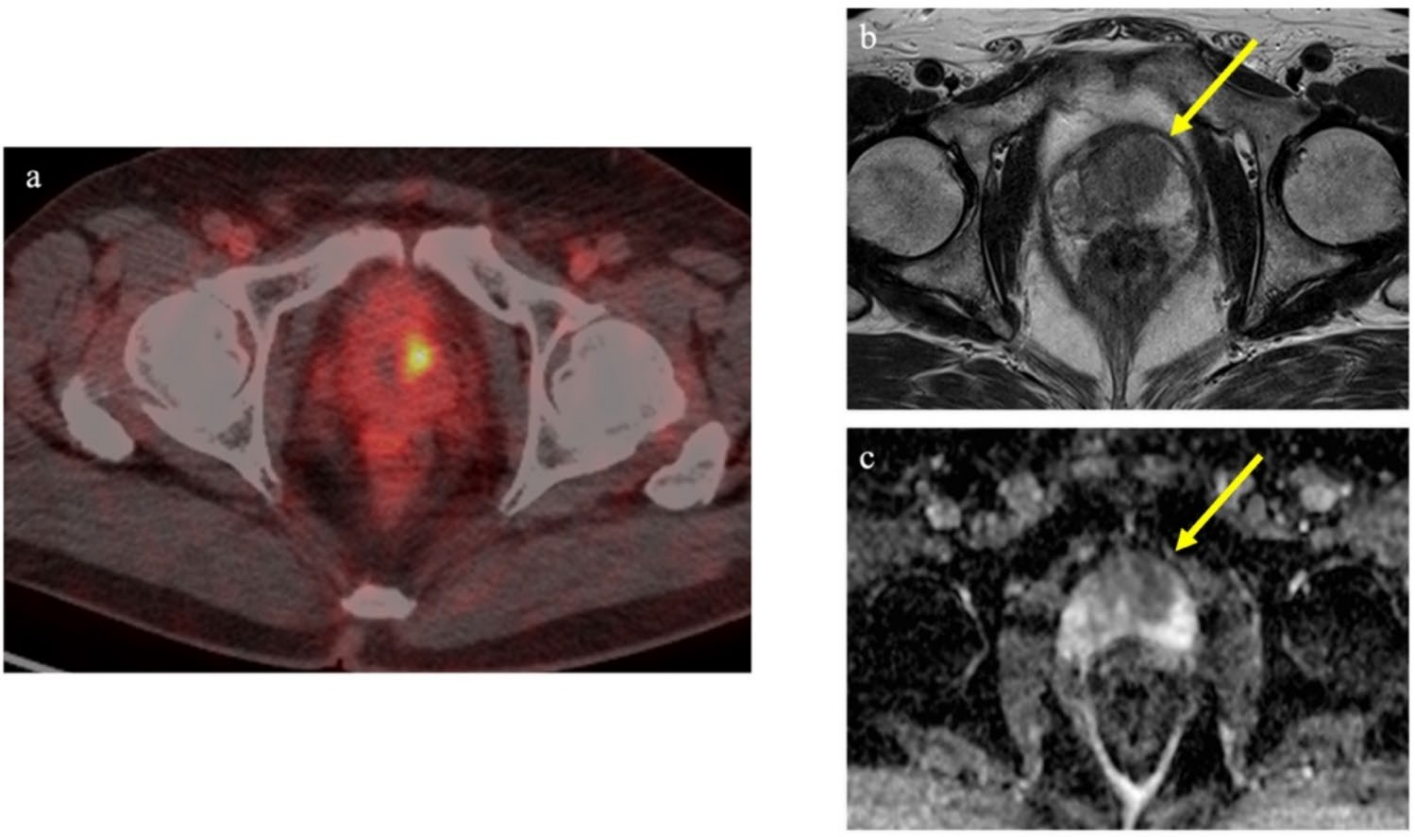
PET/CT using ^18^F-FSU-880 in a 70-year-old man with biochemical recurrence after radiation therapy (cT3aN0, Gleason score 4 + 5). PSA level increased to 2.33 ng/ml. Focal uptake was observed in the transitional zone of the prostate on the left side, suggesting local recurrence (**a**). The initial MRI (conducted 8 years earlier) showed a signal-hypointense lesion on T2-weighted imaging with restriction of water diffusion in the transitional zone on the left side, at the same site as the recurrence. **a** Transaxial PET/CT fusion image. **b** Transaxial T2-weighted MR image. **c** Transaxial ADC map

**Fig. 5:**
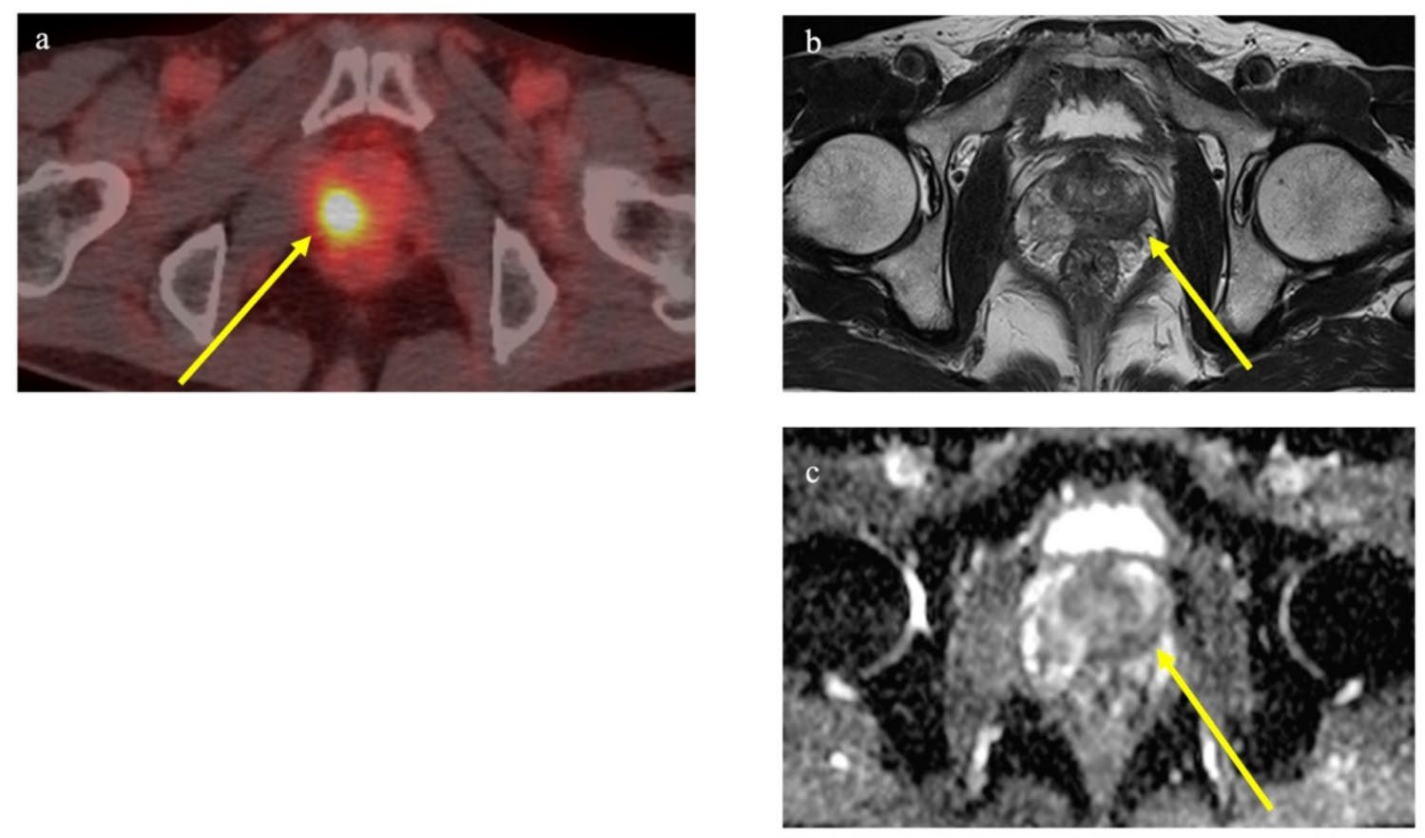
PET/CT using ^18^F-FSU-880 in a 79-year-old man with biochemical recurrence after radiation therapy (cT3bN0, Gleason score 4 + 4). PSA level increased to 4.4 ng/ml. Focal uptake was observed in the right side of the prostate, suggesting local recurrence (**a**). The initial MRI (conducted 12 years earlier) showed a signal-hypointense tumor on both T2-weighted imaging and ADC mapping in the peripheral zone of the left prostate, distinct from the recurrence site (**b**, **c**). **a** Transaxial PET/CT fusion image. **b** Transaxial T2-weighted MR image. **c** Transaxial ADC mapping

External-beam radiation therapy is a standard treatment option for non-metastatic prostate cancer. Local recurrence within the prostate after radical treatment is an important pattern of recurrence. Local recurrence was initially thought to typically occur at the same site as the primary tumor, although only few MRI studies have explored the relationship between site of local recurrence and the primary tumor [49–51]. Those studies suggested that local recurrence typically occurred in the same site as the primary tumor. An attempt has been made to examine the relationship between primary site and local recurrence site using PSMA PET/CT. Aizawa et al. utilized PSMA PET/CT to examine spatial associations between initial tumor lesions on MRI and recurrence sites on PSMA PET/CT. The findings indicated that, in two-thirds of cases, sites overlapped or were identical [52]. Figure 4 shows a case of local recurrence identified on PSMA PET/CT at the same site as the primary tumor. In response to these investigations, the effectiveness of a focal boost to the primary tumor was proposed to enhance treatment outcomes [53, 54]. The FLAME Phase III trial specifically evaluated the benefit of adding focal boost to dominant intraprostatic lesions and reported significantly better biochemical disease-free survival in the focal-boost group [5-year biochemical disease-free survival rate, 92% vs 85%; hazard ratio (HR) 0.45; *p* < 0.01] [54]. Importantly, this increase in effectiveness did not correspond with any significant increase in adverse effects. However, it should be noted that recurrence does not invariably occur at the same site as the primary tumor. Figure 5 depicts an example of recurrence at a site clearly distinct from the primary tumor.

### Application to salvage radiation therapy (SRT) after total prostatectomy

**Fig. 6:**
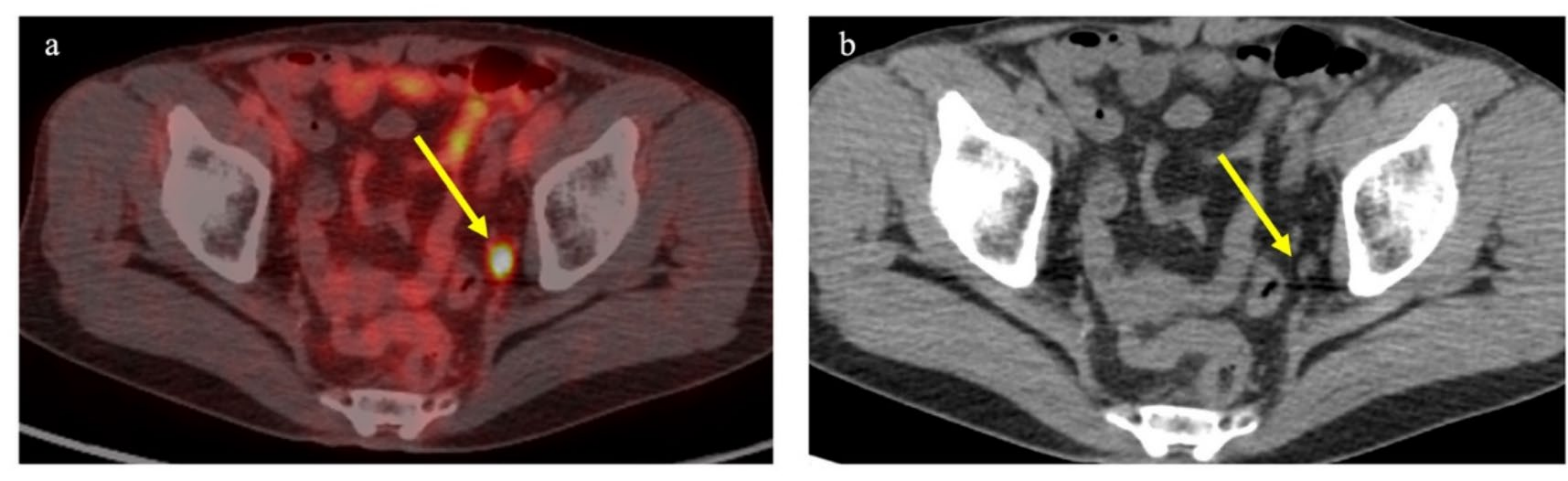
PET/CT using ^18^F-FSU-880 in a 71-year-old man with biochemical recurrence after total prostatectomy (pT2bN0, Gleason score 4 + 3). PSA level increased to 2.10 ng/ml. Focal uptake was observed in a left obturator lymph node (SUVmax; 10.2). Salvage radiation therapy had been considered before scanning, but this treatment plan was subsequently adjusted to hormonal therapy. **a** Transaxial PET/CT fusion image. **b** Transaxial CT

SRT to the prostate bed is a potentially curative treatment for patients experiencing BCR after total prostatectomy. According to the 2016 edition of the Japanese Clinical Practice Guideline for prostate cancer, SRT after total prostatectomy is a valid treatment option, is recommended with grade B, and should be initiated for PSA levels < 0.5 ng/ml [55, 56]. However, imaging identification of local recurrence prior to SRT is not deemed necessary. Despite this approach, only 50–55% of patients achieved PSA levels < 0.1 ng/ml after SRT, meaning that about half of patients cannot be cured with SRT alone [57, 58]. More appropriate case selection may thus be indispensable. Leeuwen et al. reported that in a study of 70 patients with BCR after total prostatectomy for whom SRT was planned, ^68^ Ga-PSMA-11 revealed lesions outside the prostatic fossa in 20 patients (29%); among these patients, extra pelvic lesions were observed in 4 patients [59]. These findings suggested that these patients did not achieve durable response to SRT. Figure 6 depicts a case in which SRT to the prostatic fossa was initially planned for a patient with BCR after total prostatectomy. However, PSMA PET/CT identified lymph-node metastasis in the left obturator region, leading to a change in the treatment plan to hormonal therapy. Recent attempts have been made to incorporate PSMA PET/CT in determining the indications for salvage SRT. Meijer et al. investigated outcomes among patients who underwent PSMA PET/CT prior to SRT compared to those who did not, revealing that the BCR rate at 1 year was only 8% in the group that underwent PSMA PET/CT, compared to 21% in the group without prior PSMA PET/CT [60]. This suggested that including PSMA PET/CT in the decision-making process for SRT might enable more appropriate case selection.

### Application to oligometastasis and metastasis-directed therapy (MDT)

Oligometastasis represents a condition intermediate between localized and metastatic disease, characterized by limited metastatic capacity where local treatment of metastatic sites can be effective [61, 62]. Typically defined by the presence of 3–5 or even fewer metastases, the concept of oligometastasis has gained prominence due to the effectiveness of metastasis-directed therapy (MDT). MDT is a high-intensity therapeutic approach targeting metastases, and has shown promising results in recent evaluations [63]. One purpose of MDT is to delay the initiation of palliative androgen-deprivation therapy (ADT). Phase II clinical trials have demonstrated the utility of MDT in managing oligometastases following curative treatment [64, 65]. The STOMP study compared stereotactic body radiation therapy (SBRT) (30 Gy/3 fr) or surgery against active surveillance in patients with oligometastases (≤ 3 metastases) as diagnosed by ^11^C-choline-PET/CT, and identified a significant extension in ADT-free survival (median ADT-free survival: 21 months vs 13 months, HR 0.60; *p* = 0.11) [65]. Similarly, the ORIOLE study highlighted the benefits of stereotactic ablative radiotherapy, showing improved median progression-free survival compared to observation in oligometastatic cases (≤ 3 metastases) diagnosed by the conventional imaging (not reached vs 5.8 months, HR 0.30; *p* = 0.002) [64].

**Fig. 7:**
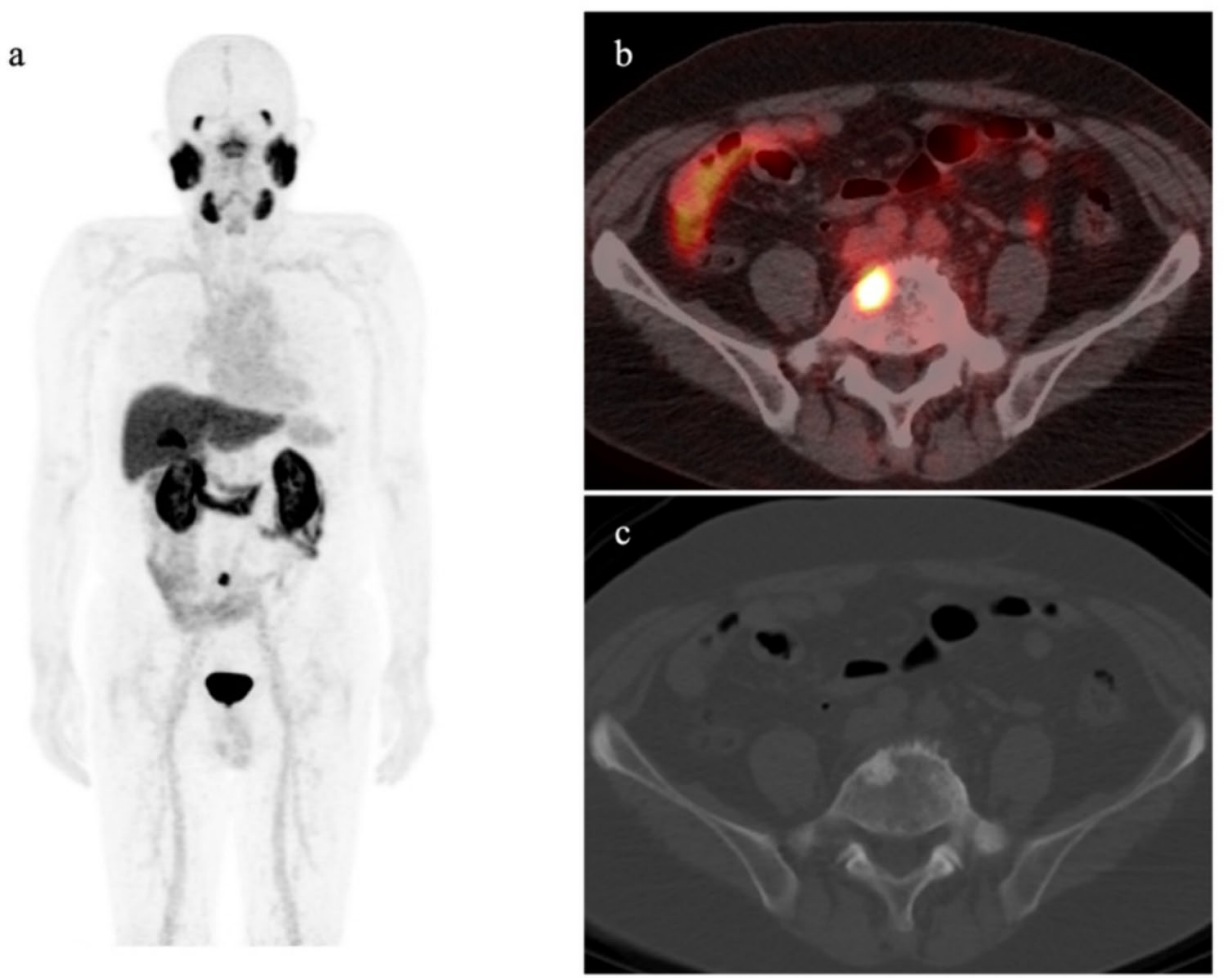
A 74-year-old man who had previously undergone total prostatectomy (pT2cN0, Gleason score 3 + 4) demonstrated biochemical recurrence. PSA level elevated to 1.44 ng/ml. PET/CT with ^18^F-FSU-880 showed focal uptake in the lumbar spine (SUVmax: 6.9), suggesting bone metastasis. As the metastasis was solitary, metastasis-directed therapy to the bone metastasis was performed, resulting in a decrease in PSA to 0.05 ng/ml. **a** Maximum intensity projection image. **b** Transaxial PET/CT fusion image. **c** Transaxial CT

Accurate diagnosis of oligometastasis necessitates confirmation that only a few metastases exist. Using diagnostic modalities with limited detection capabilities may lead to underestimation of the metastatic burden, and thus misclassification of patients. Recent studies have suggested that PSMA PET/CT, as a sensitive diagnostic modality, can more accurately identify oligometastatic cases. Cases in which stereotactic ablative radiotherapy (SABR) was administered to all PSMA PET/CT-positive lesions demonstrated significantly improved distant metastasis-free survival compared to cases where SABR was not performed (29 months vs 6 months, HR 0.19; *p* < 0.01) [64]. Figure 7 shows successful MDT to a solitary lumbar spine metastasis identified by PSMA PET/CT, resulting in the normalization of PSA levels. While the NCCN guideline described SBRT as acceptable in practice [5], the Japanese clinical practice guidelines for prostate cancer have yet to formalize the role of MDT, suggesting that application should be determined on a case-by-case basis.

## Pitfalls

PSMA PET/CT has demonstrated high sensitivity for detecting prostate cancer lesions, with a detection rate of approximately 90% at PSA values ≥ 1.0 ng/ml [20, 22, 66, 67]. However, this also means that approximately 10% of cases are PSMA-negative despite relatively high PSA values. Prostate cancer lesions may be able to be detected on ^18^F-fluorodeoxyglucose (FDG) PET/CT in cases where PSMA PET/CT shows negative results. Among 36 patients with negative results for PSMA PET/CT, FDG PET/CT detected cancer sites in six patients, with mean PSA values of 16.7 ng/ml, compared with mean PSA values of 0.8 ng/ml in the 30 patients negative for both PSMA PET/CT and FDG PET/CT [68]. One reason for the presence of a certain number of prostate cancers with negative results from PSMA PET/CT and positive results from FDG PET/CT appears to involve neuroendocrine de-differentiation resulting in the downregulation of PSMA expression and the absence of radiotracer uptake [69]. Concurrently, neuroendocrine prostate cancer resulted in higher malignancy and increased glucose metabolism compared to typical prostate cancer.

**Fig. 8:**
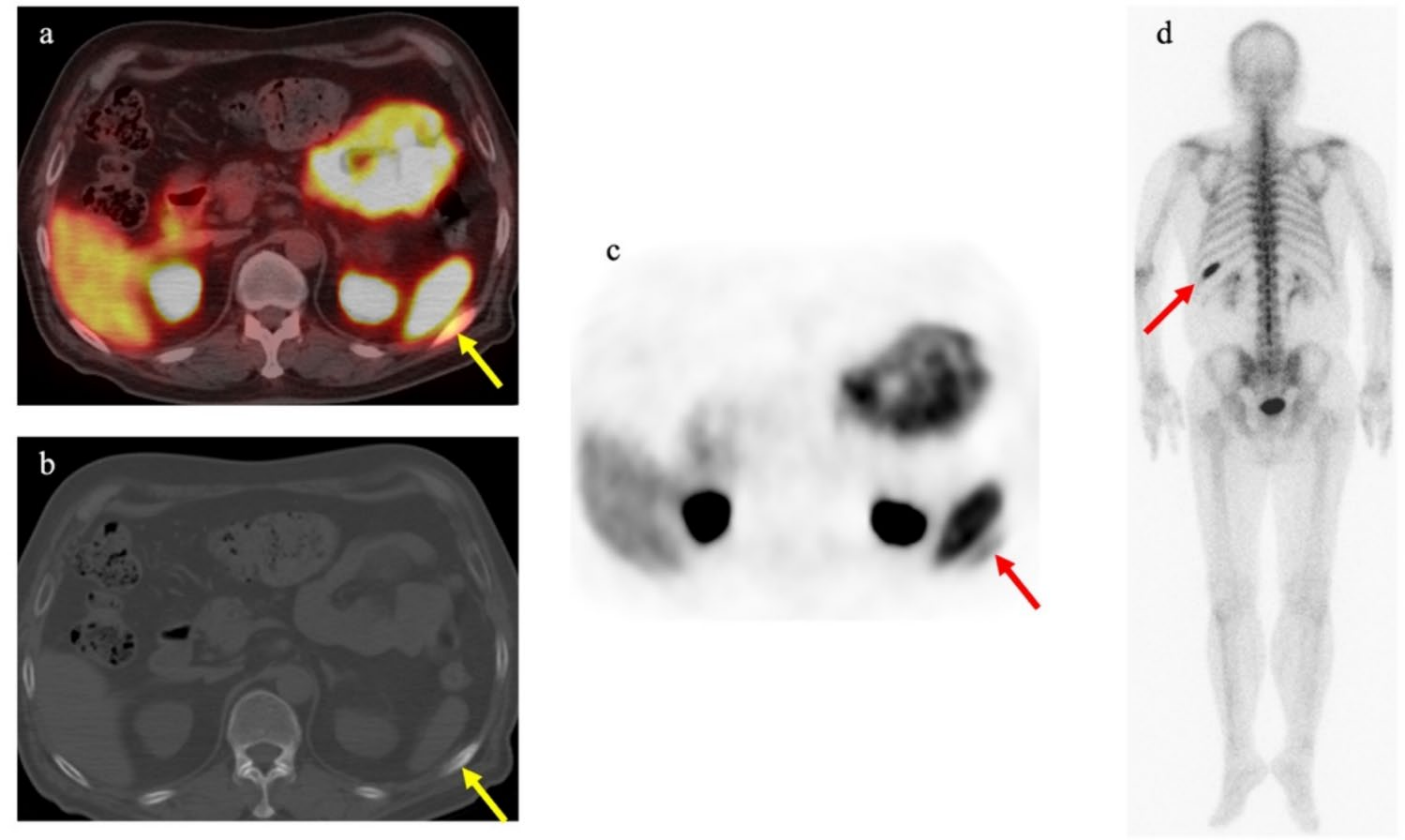
PET/CT using ^68^ Ga-PSMA-11 in a 69-year-old man with biochemical recurrence following intensity-modulated radiation therapy (cT3bN0M0, Gleason score 5 + 4). PSA level increased to 3.10 ng/ml. Focal uptake was observed in the left dorsal rib, but identification was challenging due to intense physiological uptake by the spleen. CT revealed slight sclerosis at the rib lesion site. Bone scintigraphy showed high uptake along the longitudinal axis, a typical pattern for bone metastasis. **a** Transaxial PET/CT fusion image. **b** Transaxial CT. **c** Transaxial PET. **d** Posterior view of whole-body bone scintigraphy

PSMA PET/CT shows physiologic uptake in tissues where PSMA is expressed. Knowledge about physiologic uptake is crucial for distinguishing malignant lesions from benign lesions or physiologic uptake. For tracers excreted through the urinary tract, excretion occurs in the kidneys, ureters, and bladder. High accumulation is also observed in the lacrimal, submandibular, and parotid glands [70]. The exact mechanisms remain unclear, but nonspecific secretion and expression of PSMA have been suggested [71]. PSMA is also expressed in other normal tissues, including the proximal tubules of the kidneys, duodenum, liver, spleen, and so on [72]. A case where physiological uptake in the spleen obscured the identification of rib metastasis is shown in Fig. 8.

Despite the name suggesting specificity for the prostate, PSMA is also expressed not only in prostate cancer, but also in the endothelial cells of neovascular vessels and ganglia, which can represent a pitfall in diagnostic imaging [73]. Uptake in ganglia was reportedly observed in 98% of cases using ^68^ Ga-PSMA-11 [74]. The anatomical location, low SUVmax (approximately 2–3) and band-shaped or teardrop configuration were indicative findings allowing differentiation from lymph-node metastases [74].

Since PSMA is expressed in the endothelial cells of neovascular vessels, uptake by various benign and malignant tumors such as renal cell carcinoma, hepatocellular carcinoma, hemangioma, and meningioma has been reported [75–79]. Significant abnormal uptake in locations atypical for prostate cancer metastasis should be evaluated with caution and in conjunction with the other modalities. In particular, care should be taken to differentiate between vertebral hemangioma and bone metastasis [75, 77].

Characterized by minimal excretion in the urine, ^18^F-PSMA-1007 has shown efficacy in detecting small lesions around the bladder and prostate region [15, 23, 24]. However, nonspecific uptake in bone has also been reported, requiring differentiation from bone metastases. The ribs are the most frequent site of bone uptake for this tracer (57.5%), followed by the pelvic bones (24.8%) and spine (9.7%) [80].

## Conclusion

PSMA-PET/CT scans have recently been introduced in Japan and are expected to be widely adopted. This modality offers high detection capacity for prostate cancer lesions and is anticipated to significantly impact the treatment of prostate cancer with application in various scenarios of prostate cancer treatment.
